# Development of a questionnaire investigating the physical and psychological well-being and need for rehabilitative strategies in patients with pulmonary embolism

**DOI:** 10.1186/s41687-025-00933-x

**Published:** 2025-08-13

**Authors:** Caroline Sindet-Pedersen, Jenny Bjerre, Nina Nouhravesh, Mohamad El-Chouli, Simone Hofman Rosenkranz, Morten Lamberts, Mikkel Porsborg Andersen, Erik Lerkevang Grove, Anette Arbjerg Højen, Morten Schou, Gunnar Gislason, Nina Føns Johnsen

**Affiliations:** 1 Department of Cardiology, Copenhagen University Hospital - Herlev and Gentofte, Herlev, Denmark; 2 Department of Cardiology, Nordsjaellands Hospital, Hilleroed, Denmark; 3 Department of Clinical Medicine, Faculty of Health and Medical Sciences, University of Copenhagen, Copenhagen, Denmark; 4 Department of Cardiology, Zealand University Hospital, Roskilde, Denmark; 5 The Danish Heart Foundation, Copenhagen, Denmark; 6 The Prehospital Center Region, Zealand, Næstved Denmark; 7 Department of Cardiology, Aarhus University Hospital, Aarhus, Denmark; 8 Department of Clinical Medicine, Faculty of Health, Aarhus University, Aarhus, Denmark; 9 Danish Center for Health Services Research, Department of Clinical Medicine Aalborg University, Aalborg, Denmark

**Keywords:** Patient reported outcomes, pulmonary embolism, PEmb-QoL

## Abstract

**Purpose:**

Quantitative questionnaires can provide a deeper understanding of the health-related physical and psychological well-being in patients who have experienced a pulmonary embolism (PE). This paper describes the development of a questionnaire aiming to assess physical and psychological well-being and the need for rehabilitative strategies among patients diagnosed with PE.

**Methods:**

The International Society for Quality-of-Life Research (ISO-QOL) recommendations for patient-reported outcomes were used. Conceptualization was conducted through literature review and expert interviews, followed by operationalization where items (questions) were constructed. To test content validity, the questionnaire was reviewed by experts and a series of cognitive interviews were performed. Finally, the questionnaire was distributed digitally to 82 randomly selected patients with PE in Denmark.

**Results:**

The questionnaire reached a response rate of 72%. The median age of responders was 71.5 years [inter quartile range: 64.2; 77.0], with 51.9% being female. Most patients did not feel safe about being discharged, with only 9.3% responding that they felt safe to some or a high degree. Approximately 15% were offered physical therapy, 7.9% were offered educational activities addressing psychological reactions, and 3.9% received psychological support.

**Conclusion:**

The questionnaire showed good content validity and a high response rate. The results from the questionnaire have the potential to increase focus and awareness of the potential clinical and social impact of PE. It will facilitate optimized medical interventions and guide physicians in providing appropriate follow-up care for patients with PE.

**Supplementary information:**

The online version contains supplementary material available at 10.1186/s41687-025-00933-x.

## Introduction

Questionnaire data are essential for understanding the diverse experiences of patients with pulmonary embolism (PE), a condition with varying impacts influenced by individual risk factors and demographics [1, 2]. Previous studies have primarily focused on the short-term care of patients with PE, and recent advances have improved the quality of acute treatment [3]. However, the long-term burden of PE remains insufficiently explored [4]. Many patients continue to report persistent dyspnoea, reduced physical capacity, anxiety, and depression long after the acute phase [4, 5]. Despite these findings, large-scale studies examining patient-reported outcomes (PRO) in this patient population are still lacking.

PROs are crucial for understanding the broader impact of illness from the patient’s perspective. They provide insights into symptoms, functioning, and quality of life that are not readily captured through clinical or administrative data alone [6, 7]. In the context of PE, PROs offer valuable information on how patients experience and adapt to the condition over time and can help identify needs that may otherwise go unrecognized in standard follow-up care [6]. Collecting such data is essential for guiding patient-centered clinical decision-making and for developing supportive interventions tailored to individuals’ lived experiences.

While existing instruments have primarily focused on health-related quality of life, few tools have explored the broader aspects of long-term recovery following PE [8]. To address this gap, we developed a questionnaire that not only assesses physical and psychological well-being, but also includes items on anxiety symptoms, needs for physical and psychological rehabilitation, and interest in educational support. The questionnaire is intended to provide important insights into patients’ physical and psychological well-being and rehabilitative needs, while recognizing that long-term recovery after PE is complex and that further research may refine and expand these findings.

This paper describes the development of a questionnaire aiming to assess physical and psychological well-being and the need for rehabilitative strategies among patients diagnosed with PE.

## Methods

**Figure 1:**
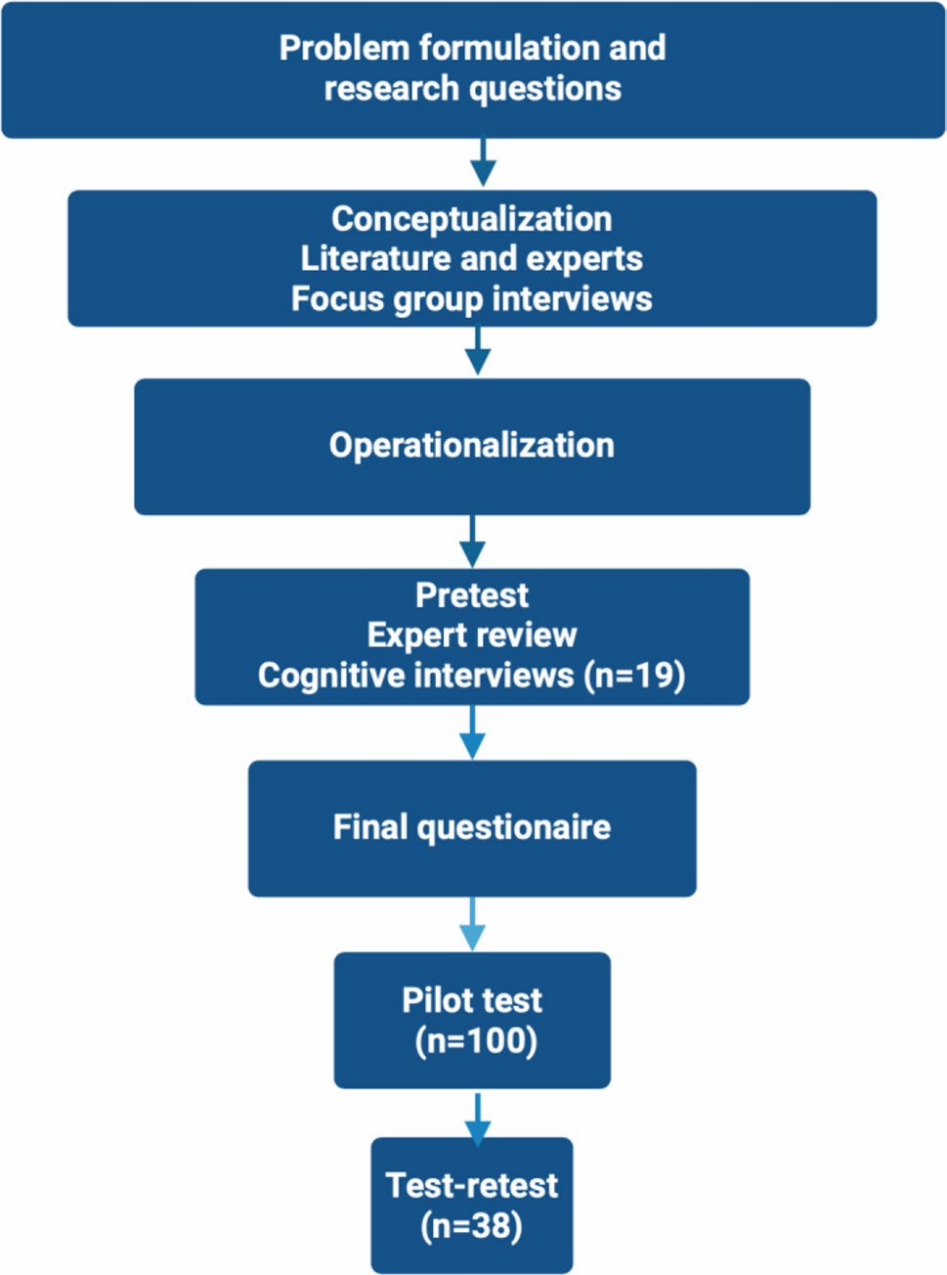
Overview of development of questionnaire

We used the International Society for Quality of Life Research (ISO-QOL) recommendations for patient-reported outcomes [9]. The recommendations were used as guidance throughout the process of developing the questionnaire and during pre-testing (Fig. 1).

## Problem formulation

We defined the following problem formulation: What are the physical and psychological outcomes for patients following a pulmonary embolism, and what are their needs for psychological, educational, and physical rehabilitation? Based on the problem formulation, three research questions were defined: 1) What are the physical consequences of a PE; 2) What are the psychological consequences of a PE; 3) What are the patients’ needs for psychological, educational, and physical rehabilitation?

## Conceptualization

Conceptualization is the concept of defining and arguing for what is measured in the questionnaire, in the following this is described a long with a description of the boundaries for the questionnaire (Table S1).

### Literature review and interview with experts

A thorough literature review was performed to identify existing literature on studies investigating the physical and psychological consequences of having experienced a PE. Several experts were interviewed about their experiences with long-term outcomes in patients following PE, including cardiology consultants, professors and nurses. In addition, the first author (CS) spent a day in the PE out-patient clinic (with ML), observing which questions the patients asked and what information they were given by the treating physician. Based on the literature review, expert interviews and observations in the outpatient clinic, a conceptual framework was developed. Through this, each concept in the problem formulation was specified, and with the research questions in mind, nine concepts were developed. A list of variables was created and, subsequently, transformed into items (questions). Supplementary Table 1 shows the questionnaire conceptualization process, including the concept, sources, variable definition, and related question. For example, the first research question regarding physical symptoms gave rise to three concepts with a varying number of corresponding variables.

### Focus group interviews

Prior to the construction of the questionnaire, semi-structured focus group interviews were conducted [10]. Focus group interviews provide in-depth knowledge into interviewees’ feelings and experiences with PE [10, 11]. Based on the conceptual framework, an interview guide was developed. Four main themes, along with corresponding subthemes, were established to guide the interviews. Participants were recruited from the outpatient clinics at Herlev and Gentofte Hospital. Interviews were performed/conducted until saturation was achieved, with no new themes emerging by the interviewees [11]. Purposeful sampling was used identifying to select participants based on their knowledge, experiences, age, sex, and provoking factors such as cancer, pregnancy, and surgery. Six interviews were conducted with a total of 17 participants.

### Choice of validated scales

The International Consortium for Health Outcomes Measurement (ICHOM) has defined a set of patient-reported outcome measures which they suggest measuring: 1) Quality of life, 2) Functional limitations, 3) Pain, 4) Dyspnea, 5) Satisfaction with treatment, 6) Psychosocial well-being, 7) Changes in life perspective. These outcome measures fit well into the conceptual framework that was developed and included into the definition of variables and items. In addition, ICHOM recommends a set of scales to measure the different outcome measures [6]. Not all recommended scales were used for this questionnaire due to concerns about questionnaire length and thus burden for the responders, potentially influencing the response rate. Table 1 depicts the outcome measures with the corresponding chosen validated scales. PEmb-QoL is a disease-specific, health-related quality-of-life questionnaire aimed specifically at patients with PE [8, 12]. On the other hand, the WHO5 questionnaire is a generic scale that measures current mental well-being [13, 14]. The WHO5 was combined with the two first questions from the Anxiety Symptom Scale (ASS) [15]. and the two first questions from the Major Depression Inventory (MDI) in order to screen for depression [16, 17]. In addition, specific questions from a large Danish nationwide questionnaire aimed at patients with cardiovascular disease (“Life with a heart disease”) [18]. were chosen for future comparison between patients with other cardiovascular diseases than PE.

**Table 1:** Validated scales, questions, and outcome measures

Scale/question	Outcome measures
**Validated scales**	
PEmb-QoL	Quality of life
WHO 5	Psychological well-being
WHO 5, ASS-2, MDI-2	Anxiety and depression
ICHOM	Life perspective
Life with a heart disease	Experiences with health care system, need for rehabilitative and educational strategies
**Questions**	
How would you rate your overall health	General health
Are you extra aware of symptoms (heart palpitations, shortness of breath, pain/tenderness in the legs) of a new blood clot in the lung?	Awareness of symptoms
“Have you experienced a change in your expectations, aspirations, values, or perspectives on life opportunities since the diagnosis of PE?”	Change in life perspective
“Do you generally feel satisfied with your blood thinning treatment of your blood clot in the lung?”	Satisfaction with treatment
“Does it bother you that you may get small bleedings (bruises, bleeding when brushing your teeth, nosebleeds and the like) from your blood thinning treatment?”	Burden of side effects
Is it a burden for you taking blood thinning treatment?	Burden of treatment
Do you feel that your blood thinning treatment limits you in your everyday life?	Limitations in everyday life because of anticoagulant treatment
Do you feel that you have received the necessary information about why you should stop taking the blood-thinning medication?	Information on ending treatment
Are you worried about no longer having to take blood thinners?	Concern about ending treatment

## Operationalization

Operationalization is the process of constructing items (questions) from the variable definitions. The general rules for valid item construction were followed [19]. The questions were designed to be suitable for the target group, easy to understand, clear, concise, and straightforward. Help text was included where deemed necessary. To minimize recall bias, three distinct time periods were defined and underscored in the questionnaire; “6 weeks prior to your diagnosis with a PE”, “in the past 4 weeks”; and “in the past 2 weeks”. These time periods were tested and deemed appropriate through the pretesting process using cognitive interviews. Factual questions had the following response category; yes/no, whereas opinion questions and questions regarding psychological well-being were constructed with a corresponding Likert scale. The scale differed depending on the questions asked. Some of the validated scales used a specific linguistic formulation and were not changed. As for the questions constructed by the research group, the same scale was used for all questions. The first question in the questionnaire was a filter question to make sure that the patients indeed had experienced a PE. The complexity of the questionnaire increased throughout the questionnaire, with background questions in the beginning followed by questions regarding quality of life and ending with a couple of easier questions regarding anticoagulation therapy.

## Content validity

To test content validity the questionnaire was reviewed by experts, followed by a series of cognitive interviews.

### Pretest: expert review

Following the construction of the questionnaire, it was carefully reviewed by an expert panel: ML, TK, and ELG (cardiology consultants), NF, BR (questionnaire experts), AH (nurse, PhD). They all independently reviewed each question and were asked to judge whether the questions were relevant in relation to the aim. They were also asked to determine whether any of the questions were sensitive and whether they were relevant to the patients. In addition, a questionnaire expert, BR, reviewed the questionnaire and commented on the scales and layout, which were adjusted accordingly. Based on the comments from the expert panel, some changes were made in selected formulations, additional risk factors and symptoms were included (questions 3 and 4) and help text was introduced where appropriate. The questions were deemed relevant according to the problem formulation. Patients also found the questions to be relevant, easy to understand, and straightforward to answer. Lastly, no questions were considered difficult (e.g. overly sensitive or inappropriate). Potentially problematic questions were chosen to be kept in the questionnaire for cognitive interviews.

### Pretest cognitive interview

The paper version of the questionnaire was tested at two hospitals in different geographic regions of Denmark: Region Hovedstaden (Herlev and Gentofte Hospital) and Region Midtjylland (Aarhus University Hospital). A total of 19 patients were included, with groups ranging from 3–5 participants. The interviews were performed over eight weeks. Verbal probing technique was used, and Tourangeau’s four stages model was used in the development of probes investigating the 1) Comprehension of the question; 2) Retrieval from memory of relevant information; 3) Decision processes; and 4) Response processes [20, 21]. The probes were either scripted or occurred spontaneously. This process was done to identify difficult questions, and to identify any hesitancy in answering the questions. After each session, head investigator (CS) evaluated the comments and summarized the results. Some alterations were made, including reordering a few questions, inserting more help text, and altering the wording of some questions.

## Pilot test

The Danish national patient register was used to identify patients diagnosed with PE between 1^st^ of January 2020 and 30^th^ of September 2022. Exclusion criteria included age under 18 and over 100, and if the patient had a diagnosis with a PE in the five years before 1^st^ of January 2020. Among these patients, 100 were randomly selected to participate in the pilot test. In Denmark, communication between the authorities and citizens occur via National Digital Post. However, not every citizen has National Digital Post and especially elderly patients will still receive official mail by physical post. Out of the randomly 100 selected patients, 18 did not have e-Boks. In the pilot test, we chose not to send out physical letters, however, when distributing the final questionnaire, those without National Digital Post received a physical letter with the questionnaire, including a return envelope. The electronic questionnaire was set up using the online survey platform SurveyXact, developed by Rambøll A/S and approved by the Capital Region of Denmark. The pilot test questionnaire was distributed to 82 patients, with two reminders sent to non-responders one week apart (Table S2). Participants were asked for permission to contact them by phone regarding either an interview about the questionnaire or to participate in a test-retest.

### Telephone interviews

Patients were interviewed using an interview guide regarding specific questions that had been identified as problematic from the cognitive interviews (Table S2). In the end, the participants were also enquired about their overall opinion of the questionnaire.

## Test-retest

A test-retest was performed to investigate the reproducibility of the questionnaire. Patients were invited to participate in the test-retest, and if they were willing to participate the test-retest questionnaire was distributed to them three weeks after receiving the first questionnaire.

## Statistical analyses

In this present study, selected items from the questionnaire were chosen to be presented from the pilot test. Of note, the number of responses to each question may vary depending on the number of patients who responded to each question. Categorical variables are presented as counts with percentages and as medians and interquartile range in case of non-normal distributed data. We assessed the test-retest reliability of questionnaire items using Cohen’s kappa, applying unweighted kappa for categorical variables and weighted kappa for ordinal variables (e.g., Likert scales). Perfect agreement across both time points was assigned a kappa of 1. When there was no variation in one time point but disagreement between test and retest, kappa was set to 0, as the consistency of responses could not be meaningfully assessed. For example, in item *sp03_d*, responses varied at test but were identical at retest, resulting in a kappa of 0. Confidence intervals were calculated by bootstrap resampling (1,000 iterations). Kappa values were categorized into standard interpretation ranges (poor, fair, moderate, substantial, and almost perfect) [22]. All analyses were performed using R (version 4.2.1 for Windows, R Foundation for Statistical Computing).

## Ethics

The data-responsible institution (Capital Region of Denmark) approved the study (approval number P-2021-684).

## Results

## Focus group interviews

The concepts identified through the literature review and expert interviews were confirmed. Several new concepts emerged during the interviews. A key finding was patients’ lack of understanding regarding the absence of control/follow-up CT-scans after PE, with a strong wish for such scans to confirm resolution. Age and gender differences were also evident: younger patients were more sceptical toward the healthcare system, while older patients were generally more satisfied with care. Women reported more symptoms than men, with older men attributing symptoms to aging rather than PE. Across all patients, there was confusion about the follow-up process and uncertainty after discharge.

## Pilot test

### Response rate

Of the 82 eligible patients, 80 were alive at questionnaire distribution. Among them, 75 patients were eligible (5 indicated no PE diagnosis). A total of 54 patients responded, yielding a response rate of 72% (Fig. 1).

### Patient characteristics

The median age of responders was 71.5 years [inter quartile range: 64.2; 77.0], with 51.9% being female and 94.3% had experienced a first-time PE. (Table 2). A total of 51.8% had experienced a risk factor for venous thromboembolism six weeks before PE diagnosis, with the most common being deep venous thrombosis (18.5%), recent hospitalization (14.8%), and cancer (13.0%). Overall, 75.9% reported symptoms prior to diagnosis, most commonly tiredness (48.1%), dyspnoea (37.0%), and dizziness (25.9%)

**Table 2:** Baseline characteristics, risk factors and symptoms experienced and answered by the patients in the questionnaire

Patient characteristics	Total (%)
Age (Median)	71.5 [64.2, 77.0]
Sex (male)	28 (51.9)
*Number of times experienced a PE*	
1 time	50 (94.3)
2 times	3 (5.7)
More than 2 times	0 (0.0)
*Risk factors for PE experienced up to 6 weeks prior to diagnosis*	
Hip or lower limb fracture	0 (0.0)
Recent surgery	3 (5.6)
Admission with infection	6 (11.1)
Covid-19	7 (13.0)
Longer admission (>3 days)	8 (14.8)
Longer travel	2 (3.7)
Pregnancy or post-partum	0 (0.0)
Fertility or hormone treatment	1 (1.9)
Cancer	7 (13.0)
Chemotherapy	4 (7.4)
Previous deep venous thromboembolism	10 (18.5)
None of the above	25 (46.3)
Dont know	1 (1.9)
*Symptoms experienced up to 6 weeks prior to diagnosis*	
Dyspnea	20 (37.0)
Chest pain	9 (16.7)
Tiredness	26 (48.1)
Dizziness	14 (25.9)
Heart palpitations	9 (16.7)
Syncope	5 (9.3)
Pain, swelling or soreness in legs	12 (22.2)
None of the above	11 (20.4)
Do not know	2 (3.7)

PE: Pulmonary embolism

### Information and offers received

**Figure 2:**
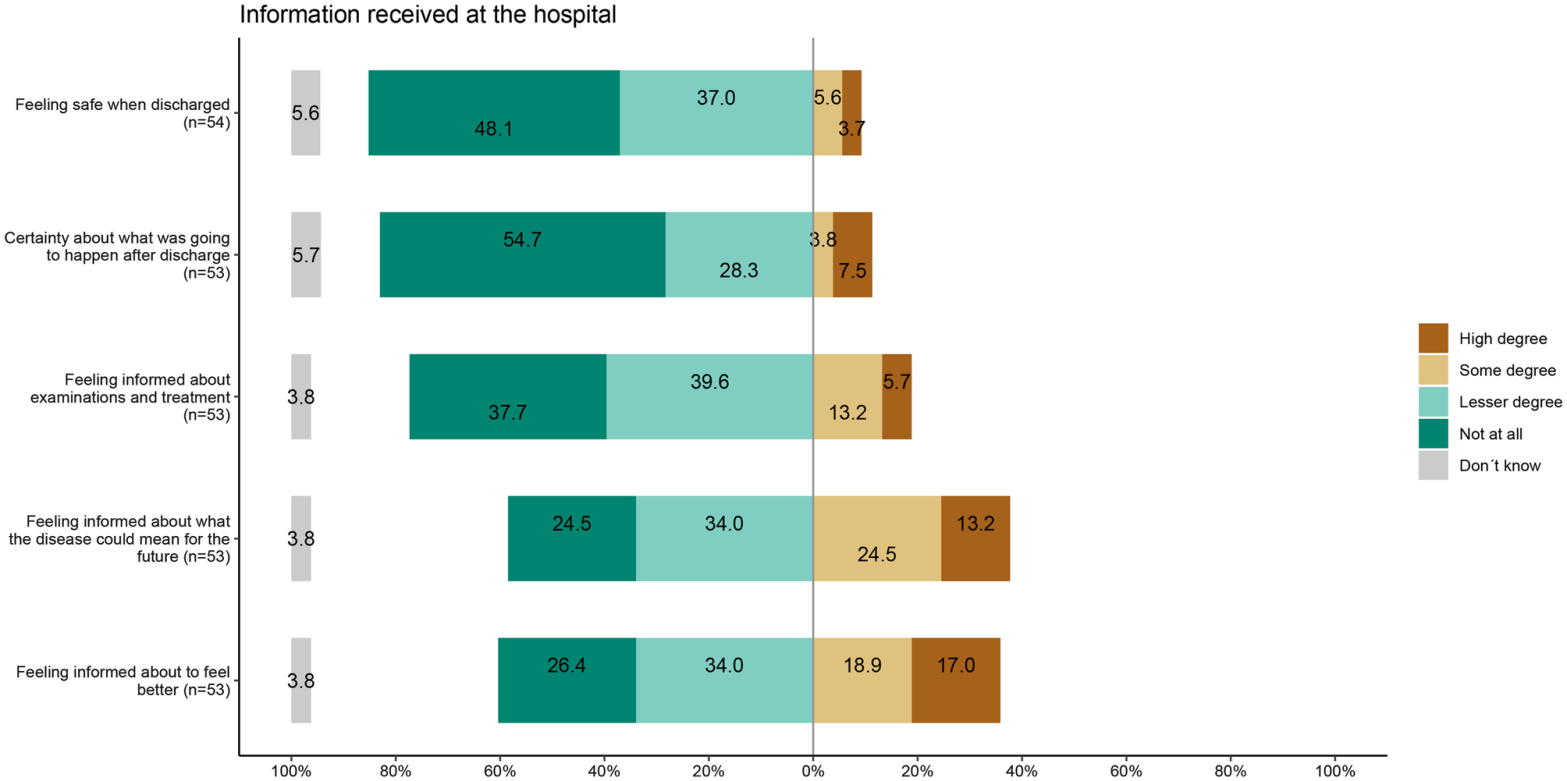
Likert plot on information received at the hospital when discharged. Included items from top to bottom are; Number 5, 6, 9a, 9b, and 9c

**Figure 3:**
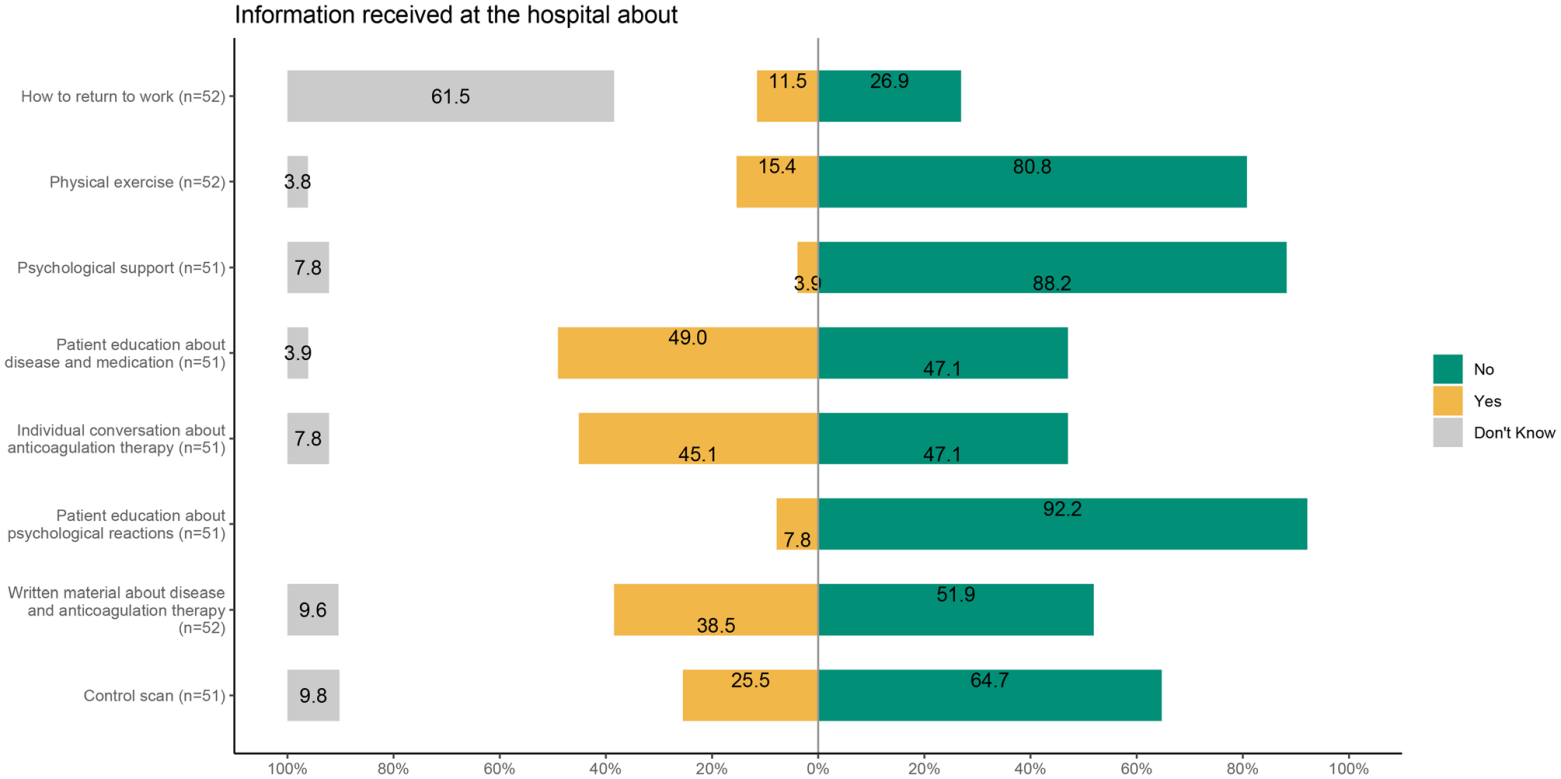
Likert plot showing the advice, support and offers of information received at the hospital when discharged. Included item from top to bottom are: 10a, 10b, 10c, 10d, 10e, 10h

Only 9.3% felt safe at discharge (Fig. 2). Most patients reported insufficient information about follow-up visits (77.3%), disease implications (58.5%), or self-care strategies (60.4%) (Fig. 2). Regarding rehabilitation offers, 50% were offered educational sessions on illness and anticoagulation, and 45% were offered an individual anticoagulation consultation (Fig. 3). Fewer received offers of physical therapy (15%), education on psychological reactions (7.9%), or psychological support (3.9%). Notably, 25% had received a follow-up control scan.

Only 9.3% felt safe at discharge (Fig. 2). Most patients reported insufficient information about follow-up visits, disease implications, or self-care strategies (Fig. 3). Regarding rehabilitation offers, 50% were offered educational sessions on illness and anticoagulation, and 45% were offered an individual anticoagulation consultation. Fewer received offers of physical therapy (15%), education on psychological reactions (7.9%), or psychological support (3.9%). Notably, 25% had received a follow-up control scan.

### Telephone interviews

A total of 15 pilot test participants were interviewed about the questionnaire. Based on their feedback, one question was removed. Overall, participants found the questionnaire relevant and the length appropriate.

## Test-retest

Of the 34 patients invited to participate in a test-retest, 18 (53%) responded. The test-retest analysis showed 61.3% moderate to perfect agreement: 11.3% of items had perfect agreement, 21.7% substantial agreement, and 28.3% moderate agreement (Table S3). Items from PEMb-QoL, WHO-5, MDI, ASS, and sections on symptoms, risk factors, and anticoagulation showed higher agreement than items derived from *Life with Heart Disease*.

## Discussion

Through a rigorous developmental process and testing, we have constructed a questionnaire aiming at investigating the physical and psychological well-being and the need for rehabilitative strategies among patients diagnosed with PE. Through expert review and cognitive interviewing, the questionnaire showed good content validity. The pilot test showed high response rate of 72%.

The pilot test reached a response rate of 72% within three weeks and after two reminders. This suggests that the participants were willing to answer the questionnaire, possibly because PE patients feel unsafe when discharged and continue to have symptom long after their discharge. Several measures were taken to increase response rates: all letters, both via e-mail and by regular mail, were addressed to the individual patients by name, the questionnaire was kept as short as possible (taking the patients 20–30 minutes to answer) by only including items that were deemed relevant, a number of validated scales were used, and cognitive testing was performed, all factors known to increase response rate [22, 23].

Surprisingly, the pilot survey/test showed that 85% of the patients to a high or some degree felt unsafe when discharged, while most patients did not feel certainty about what was going to happen, nor had they received sufficient information. These results emphasize the need for comprehensive information given at discharge for these patients. In addition, only 50% of patients were offered rehabilitative strategies, although it has been shown that rehabilitation can decrease the symptom burden [24]. Consistent with these findings, the focus group interviews also revealed that younger patients in particular expressed scepticism toward the healthcare system and uncertainty about the follow-up process, further highlighting the importance of improving communication and trust-building efforts at discharge. The observed differences in trust toward the healthcare system between younger and older patients suggest that age-specific approaches may enhance post-PE care. Older adults generally report higher trust in healthcare providers, which is associated with better satisfaction and outcomes [25]. In contrast, younger patients often show greater scepticism toward medical advice and are more likely to seek independent health information online [26]. These variations imply that follow-up strategies should be tailored: younger patients may benefit from detailed explanations, shared decision-making, and digital resources, while older patients may respond well to clear communication and consistent provider relationships.

Test-retest analysis showed 60% moderate to perfect agreement. However, questions with lower agreement were those regarding offers of rehabilitation or educational activities. A review of the responses revealed that patients often changed their answers between the initial and retest questionnaires, switching from reporting no offers of rehabilitation or education to indicating that they had received such offers. This likely occurred because patients recently diagnosed with a PE might not have received these offers yet but had received them by the time of the retest. Thus, even though three weeks were considered sufficient to avoid retention and to avoid changes in the illness, as was observed for the sufficient agreement with the PEMb-QoL, WHO5, MDI and ASS, it may have been too many questions about rehabilitation offers. Thus, for the analyses of the final questionnaire it is important to account for and stratify data based on the time elapsed since PE diagnosis.

Some limitations apply. First, we decided to only distribute the pilot test questionnaire electronically. This must be kept in mind when analyzing data from the final questionnaire, which was distributed both electronically and by regular mail. Secondly, information about the patients’ comorbidities and time since diagnosis was not available as the data was not linked to the Danish nationwide registries. Thirdly, due to the lack of linkage with register data, we were unable to compare responders with non-responders. And lastly, patients responding to the questionnaire were chosen based on registry data using an ICD-10 code for PE. The diagnosis code has a positive predictive value of 90% (Confidence interval; 78% to 96%) [27]. This means that the questionnaire could potentially be sent to a person who in fact had not experienced a PE, which was observed in the pilot test, where 6.2% responded they had not experienced a PE. Fourth, although our questionnaire includes validated items from *Life with Heart Disease* and was developed with input from experts, literature, and patients, it may not fully capture all rehabilitative needs of PE patients. Some support needs may have been overlooked, particularly those less documented in current literature [2]. Future work should assess the questionnaire’s completeness and explore whether additional items are needed to reflect the full recovery experience in a PE population.

## Conclusion

This questionnaire specifically aimed at investigating the psychological and physical well-being and rehabilitative needs in patients with PE, showed good content validity and demonstrated a high response rate. When the questionnaire has been distributed to 7500 Danish residents, it will be the largest questionnaire conducted in this patient population. From pilot testing, we anticipate a sufficient response rate and have also demonstrated fair content validity. The results from the questionnaire have the potential to increase focus and awareness of the potential clinical and social impact of PE, and will facilitate optimized medical interventions and guide physicians in providing appropriate follow-up care for patients with PE.

## Electronic supplementary material

Below is the link to the electronic supplementary material.

Supplementary material 1

## Data Availability

The data underlying this article cannot be shared publicly due to the privacy of individuals that participated in the study and due to GDPR. All data is kept in a closed folder with login on the hospital computer drive.
