# Supplementary material for: Development of a questionnaire investigating the physical and psychological well-being and need for rehabilitative strategies in patients with pulmonary embolism
Source: J Patient Rep Outcomes. 2025 Aug 13;9:101. doi: 10.1186/s41687-025-00933-x (PMC12350868; doi:10.1186/s41687-025-00933-x)
Supplement: Supplementary file 1 — Supplementary material 1 [file 41687_2025_933_MOESM1_ESM.docx]

# Supplementum

## Supplementary Table 1

Table S1: Questionnaire Conceptualization Process: The research aims, hypotheses, concepts and corresponding variable definitions

| **Aim 1: To investigate the physical consequences of having experienced a pulmonary embolism** | | | |
| --- | --- | --- | --- |
|  | **Sources** | **Variable definition** | **Question (Q)** |
| ***Concept 1: Physical symptoms experienced by the patients*** | | | |
| *Patients with pulmonary embolism experience a variety of symptoms after their pulmonary embolism* | Literature  PEMbQOL  Expert opinion  Focus groups | Whether the patient experiences pain, pressure, nagging or breathlessness | Q12 |
|  |  | Whether there is a difference in the time of day where the symptoms are experienced | Q13 |
|  |  | Whether the patients experience varying degree of pain following pulmonary embolism | Q18 |
|  | PEMbQOL | Whether the patients experience improvement in symptoms | Q14 |
|  | PEMbQOL | How much breathlessness the patient experiences | Q19 |
|  | Focus group, litterature | Whether the patients were more aware of their body and symptoms after their pulmonary embolism | Q22 |
|  | PEMbQOL | Whether the symptoms influence their social life | Q17 |
| ***Concept 2: Impact of pulmonary embolism on daily life*** | | | |
| *Patients with pulmonary embolism experience limitations in their everyday life* | Literature  PEMbQOL  Expert opinion  Focus groups | Whether the patients experience limitations in their everyday life | Q15/16 |
| ***Concept 3: Impact on work life*** | | | |
| *Patients with pulmonary embolism experience limitations in their work life* | Literature  PEMbQOL  Expert opinion  Focus groups | Whether the patients experience limitations in their work life | Q15/Q16 |
| **Aim 2: To investigate the psychological consequences of having experienced a pulmonary embolism** | | | |
| ***Concept 5: Social and emotional impact*** | | | |
| *Patients with pulmonary embolism experience social limitations* | Literature  PEMbQOL  Expert opinion  Focus groups | Whether the patients have felt like a burden to their family/friends | Q20g |
|  |  | Whether the patients have felt restricted traveling or going on trips | Q20i |
|  |  | Whether the patient feels afraid of being alone. | Q20f |
| *Patients with pulmonary embolism experience emotional complaints* | Literature  PEMbQOL  Expert opinion  Focus groups | Whether the patients had concerns about ending anticoagulation therapy. | Q20c |
|  | Literature  PEMbQOL  Expert opinion  Focus groups | Whether the patients had concerns about experiencing a recurrent event | Q20a |
|  | PEMbQOL | Whether the patient has felt emotional, depressed or irritable | Q20b/d/e |
|  | WHO5 | The emotional wellbeing of the patient | Q24 |
|  | MDI-2/ASS-2 | To which degree the patients experience anxiety and depression | Q25 |
| **Concept 6 General health perceived by the patients** | | | |
| *Patients with pulmonary embolism do not consider themselves sick* | Literature  PEMbQOL  Expert opinion  Focus groups  “life with a heart disease” | The general health perceived by the patient | Q23  Q14 |
|  |  | How the patients perceive the severity of their pulmonary embolism | Not included |
| *Patients with pulmonary embolism experience changes in life view following their pulmonary embolism* | ICHOM | Whether the patients experienced a change in life view following their pulmonary embolism | Q21 |
| **Aim 3: To investigate the patients’ need for psychological, educational and physical rehabilitation** | | | |
| **Concept 7: Information given by health care providers following pulmonary embolism regarding the time after their PE** | | | |
| *Not all pulmonary embolism patients are given proper information about their illness – including follow-up visits, reason for examinations/no examination, reason for continueing/ending anticoagulation therapy* | Expert opinion  Focus groups  “Livet med hjertesygdom” | Whether the patient felt safe about being discharged | Q5 |
|  |  | Whether the patient knew what was going to happen after they were discharged | Q6 |
|  |  | Whether the healtcare personal used a language that the patients could understand | Q7 |
|  |  | Whether the patient received the needed information regarding follow-up visits/examinations | Q8 |
|  |  | Whether the patients felt informed about the follow-up visits, what the disease could mean for their future or what they could do themselves to feel better | Q9 |
|  |  | Whether the patients understood why no control scan is offered | Not included |
| **Concept 8: The patients’ need for physical and emotional rehabilitation** | | | |
| *Not all pulmonary embolism patients were offered rehabilitation, including for example physical therapy, emotional support, patient education* | Expert opinion  Focus groups  “Livet med hjertesygdom” | How to best return to work | Q10a |
|  |  | Whether the patient was offered physical therapy | Q10b |
|  |  | Whether the patient was offered psychological support | Q10c |
|  |  | Whether the patient was offered educational activities | Q10d |
|  |  | Whether the patient was offered individual consultations about medication | Q10e |
|  |  | Whether the patient was offered educational activites regarding understanding the psychological reactions towards the disease | Q10f |
|  |  | Whether the patients were offered written material about the illness and anticoagulation therapy | Q10g |
|  |  | Wether the patients were offered a control scan | Q10H |
| *Many patients would have liked to be offered physical and emotional rehabilitation* | Expert opinion  Focus groups  “Livet med hjertesygdom” | Whether the patient would have liked to be offered physical and emotional rehabilitation | Q11 a-f |
| **Concept 8: Information about medication** | | | |
| *Patients with pulmonary embolism consider the medication a “life saver”, and not all patients understood why they had to discontinue their anticoagulation therapy* | Litterature  Focus groups | If aswer is yes to taking medication :  Whether the patients were in treatment with an anticoagulant | Q26/Q27Q28/Q29 |
|  |  | If aswer is No to taking medication:  Whether the patients understood why they had to discontinue/continue medication | Q30/Q31 |
| *Satisfaction with medication* | ICHOM | Whether the patients are satisfied with their treatment | Q26 |

PEMbQOL: Pulmonary Embolism Quality of life questionnaire, WHO5: World Health Organization-5, ASS-2 Anxiety Symptom Scale-2/MDI-2 Major Depression Inventory-2

## Table S2: Questionnaire

**Questionnaire for patients who have had a blood clot in the lung***The first question is to ensure that you are the target group for the questionnaire, as the Danish registers may well contain errors*

|  | Yes | No, I never had  a blood clot in the lungs |  |
| --- | --- | --- | --- |
| 01) During 2020, 2021 or 2022, have you been diagnosed with a blood clot in the lung? | ❑ | ❑ |  |

**You and your blood clot in the lung***The next questions are about you and your blood clot in the lung. If you have had a blood clot in the lung several times, please think back to the first time you had a blood clot in the lung in the period 2020-2022.*

|  | 1 time | 2 times | More than two times |
| --- | --- | --- | --- |
| 02) How many times have you been diagnosed with a blood clot in the lung in the period 2020-2022? | ❑ | ❑ | ❑ |

|  | *(you are welcome to tick more than one)* |
| --- | --- |
| 03) In the period 6 weeks before the diagnosis with your blood clot in the lung, did you experience any of the following? | |
| ❑ | Having broken a hip or lower leg |
| ❑ | Having surgery |
| ❑ | Being admitted due to e.g. pneumonia, |
| ❑ | Having Covid-19 |
| ❑ | Being admitted for a longer period (more than 3 days) |
| ❑ | Been on a flight or long-term seated transport |
| ❑ | Being recently pregnant or having given birth |
| ❑ | Having been in fertility treatment, hormone treatment or taken birth control pills |
| ❑ | Having Cancer |
| ❑ | Having recieved chemotherapy |
| ❑ | Having had a blood clot in the leg |
| ❑ | None of the above |
| ❑ | Do not know |

|  | *(you are welcome to tick more than one)* |
| --- | --- |
| 04) In the period 6 weeks before your diagnosis with your blood clot in the lung, did you experience one or more of the following symptoms? | |
| ❑ | Shortness of breath |
| ❑ | Pain the chest |
| ❑ | Tiredness |
| ❑ | Dizziness |
| ❑ | Heart palpitations |
| ❑ | Fainting |
| ❑ | Pain, swelling, tenderness in the leg |
| ❑ | None of the above |
| ❑ | Dont know |

**Information about blood clots in the lungs**

*The next questions are about your experience and the information you received from the hospital when you were discharged after your first blood clot in the lung in the period 2020-2022.*

|  | Yes, to a high extent | Yes, to some extent | To a lesser extent | No, not  at all | Dont know |
| --- | --- | --- | --- | --- | --- |
| 05) Were you comfortable coming home from the hospital after your first hospitalization with a blood clot in the lung? | ❑ | ❑ | ❑ | ❑ | ❑ |
| 06) Did you know what was going to happen after you were discharged from the hospital? Eg. whether you should contact your own doctor, or whether you would receive a letter from the hospital for a check-up in the ambulatory. | ❑ | ❑ | ❑ | ❑ | ❑ |

|  | To a great extent | To a high extent | To some extent | To a lesser extent | Not at all | Dont know/not relevant |
| --- | --- | --- | --- | --- | --- | --- |
| 07) Did the healthcare staff use a language that was understandable (not professional language, but comprehensible Danish)? | ❑ | ❑ | ❑ | ❑ | ❑ | ❑ |
| 08) Overall, did you get the oral and written information from the hospital staff that you needed? | ❑ | ❑ | ❑ | ❑ | ❑ | ❑ |

|  | Yes, to a high extent | Yes, to some extent | To a lesser extent | No, not  at all | Ved ikke |
| --- | --- | --- | --- | --- | --- |
| 09) Do you feel informed about the following without having to seek out/find the information yourself: |  |  |  |  |  |
| 1. What examinations and treatment courses did you have to go through? | ❑ | ❑ | ❑ | ❑ | ❑ |
| 1. What the disease can mean for your future? | ❑ | ❑ | ❑ | ❑ | ❑ |
| 1. What you can do yourself to feel better, e.g. by physical training? | ❑ | ❑ | ❑ | ❑ | ❑ |

## Advice, support and offers *The next questions concern the advice, support and offers you have received in connection with your blood clot in the lung (either at the hospital, with your own doctor or your municipality).*

|  |  | Yes |  | No |  | Dont know/not relevant |
| --- | --- | --- | --- | --- | --- | --- |
| 10) In connection with your blood clot in the lung, have you been offered the following?: |  |  |  |  |  |  |
| 1. How you can best return to the labor market |  | ❑ |  | ❑ |  | ❑ |
| 1. Physical exercise |  | ❑ |  | ❑ |  | ❑ |
| 1. Psychological support (therapist course, psychological help) |  | ❑ |  | ❑ |  | ❑ |
| 1. education about your illness and the blood-thinning medication |  | ❑ |  | ❑ |  | ❑ |
| 1. Individual education about blood thinners |  | ❑ |  | ❑ |  | ❑ |
| 1. Education in psychological reactions |  | ❑ |  | ❑ |  | ❑ |
| 1. Offer of written material describing the disease and the blood-thinning medication |  | ❑ |  | ❑ |  | ❑ |
| 1. Offer about control scan (CT scan) to see if the blood clot is gone |  | ❑ |  | ❑ |  | ❑ |

|  |  | Yes |  | No |  | Dont know/not relevant |
| --- | --- | --- | --- | --- | --- | --- |
| 11) Would you have liked the following to have been offered?: |  |  |  |  |  |  |
| 1. How you can best return to the labor market |  | ❑ |  | ❑ |  | ❑ |
| 1. Physical exercise |  | ❑ |  | ❑ |  | ❑ |
| 1. Psychological support (therapist course, psychological help) |  | ❑ |  | ❑ |  | ❑ |
| 1. education about your illness and the blood-thinning medication |  | ❑ |  | ❑ |  | ❑ |
| 1. Individual education about blood thinners |  | ❑ |  | ❑ |  | ❑ |
| 1. Education in psychological reactions |  | ❑ |  | ❑ |  | ❑ |
| 1. Offer of written material describing the disease and the blood-thinning medication |  | ❑ |  | ❑ |  | ❑ |
| 1. Offer about control scan (CT scan) to see if the blood clot is gone |  | ❑ |  | ❑ |  | ❑ |

## Well-being after your blood clot in the lung

*These questions are about your lungs. The information you give should describe how you feel. You can also indicate how capable*

*you are of carrying out your normal activities.*

|  | Every day | Several times a week | About once a week | Less than once a week | Never |
| --- | --- | --- | --- | --- | --- |
| 12) During the past 4 weeks, how often have you had any of the following symptoms from your lungs? (Circle 1 answer on each line) |  |  |  |  |  |
| 1. Pain behind or between the shoulder blades? | ❑ | ❑ | ❑ | ❑ | ❑ |
| 1. Pain on or in the chest? | ❑ | ❑ | ❑ | ❑ | ❑ |
| 1. Pain in the back? | ❑ | ❑ | ❑ | ❑ | ❑ |
| 1. Sensation of pressure? | ❑ | ❑ | ❑ | ❑ | ❑ |
| 1. Feeling that there is still “something” there? | ❑ | ❑ | ❑ | ❑ | ❑ |
| 1. “Burning sensation” in the lungs? | ❑ | ❑ | ❑ | ❑ | ❑ |
| 1. “Nagging feeling2 in the lungs? | ❑ | ❑ | ❑ | ❑ | ❑ |
| 1. Difficulty in breathing or breathlessness? | ❑ | ❑ | ❑ | ❑ | ❑ |

|  | On waking | At mid day | At then end of the day | At night | At any time of the day | Never |
| --- | --- | --- | --- | --- | --- | --- |
| 13) At what time of day are your **lung symptoms** most intense? (circle one answer) | ❑ | ❑ | ❑ | ❑ | ❑ | ❑ |

|  | Much better now than 1 year ago | Somewhat better now than 1 year ago | About the same now as 1 year ago | Somewhat worse now than 1 year ago | Much worse now than 1 year ago | I did not have any problems with my lungs |
| --- | --- | --- | --- | --- | --- | --- |
| 14) Compared to 1 year ago, how would you rate the **condition** of your **lungs** in general now? (circle one answer) | ❑ | ❑ | ❑ | ❑ | ❑ | ❑ |

|  | YES, Limited a Lot | YES, Limited a Litle | NO, Not Limited  At All | I do not work |
| --- | --- | --- | --- | --- |
| 15) The following items are about activities that you might do in a typical day. Do your lung symptoms now limit you in these activities? If so, how  Much? |  |  |  |  |
| 1. **Daily activities at work** | ❑ | ❑ | ❑ | ❑ |
| 1. **Daily activities at home** (e.g. housework, ironing,doing odd jobs/repairs around the house, gardening etc…) | ❑ | ❑ | ❑ |  |
| 1. **Social activities** (Such as traveling, going to the cinema, parties, shopping) | ❑ | ❑ | ❑ |  |
| 1. **Vigorous activities,** such as running, lifting heavy objects, participating in strenuous sports | ❑ | ❑ | ❑ |  |
| 1. **Moderate activities,** such as moving a table, hoovering, swimming or cycling | ❑ | ❑ | ❑ |  |
| 1. Lifting or carrying groceries | ❑ | ❑ | ❑ |  |
| 1. Climbing **several** flights of stairs | ❑ | ❑ | ❑ |  |
| 1. Climbing **one** flight of stairs | ❑ | ❑ | ❑ |  |
| 1. Bending, kneeling squatting | ❑ | ❑ | ❑ |  |
| 1. Walking **more than half a mile** | ❑ | ❑ | ❑ |  |
| 1. Walking **a couple of hundred years** | ❑ | ❑ | ❑ |  |
| 1. Walking **about one hundred yeards** | ❑ | ❑ | ❑ |  |
| 1. Wahing or dressing yourself | ❑ | ❑ | ❑ |  |

|  | YES | NO |  |
| --- | --- | --- | --- |
| 16) During the past 4 weeks, have you had any of the following problems with your work or other regular daily activities as a result of your lung  symptoms? |  |  |  |
| 1. Cut down the **amount of time** you spent on work or other activities | ❑ | ❑ |  |
| 1. **Accomplished** less than you would like | ❑ | ❑ |  |
| 1. Were limited in the **kind** of work or other activities | ❑ | ❑ |  |
| 1. Had **difficulty** performing the work or other activities (e.g. it took extra effort) | ❑ | ❑ |  |

|  | Not at all | Slightly | Moderately | Quite a bit | Extremely |
| --- | --- | --- | --- | --- | --- |
| 17) During the past 4 weeks, to what extent have your **lung symptoms** interfered with your normal social activities with family, friends, neighbours or groups? | ❑ | ❑ | ❑ | ❑ | ❑ |

|  | None | Very slight | slightly | Quite a bit | Serious | Very serious |
| --- | --- | --- | --- | --- | --- | --- |
| 18) How much **pain around your shoulder blades/pain in your chest** have you experienced during the past 4 weeks? | ❑ | ❑ | ❑ | ❑ | ❑ | ❑ |
| 19) How much breathlessness have you experienced in the past 4 weeks? | ❑ | ❑ | ❑ | ❑ | ❑ | ❑ |

*These questions are about how you feel and how things have been with you during the past 4 weeks as a result of your lung symptoms. For each question, please give the one answer that comes closest to the way you have been feeling.*

|  | All of the time | Most of the time | A good bit of the time | Some of the time | A little of the time | None of the time |
| --- | --- | --- | --- | --- | --- | --- |
| 20) *How much of the time during the past 4 weeks?* |  |  |  |  |  |  |
| 1. Were you worried about having another pulmonary embolism? | ❑ | ❑ | ❑ | ❑ | ❑ | ❑ |
| 1. Did you feel irritable? | ❑ | ❑ | ❑ | ❑ | ❑ | ❑ |
| 1. Would you have been worried if you had to stop taking anticoagulant medication? | ❑ | ❑ | ❑ | ❑ | ❑ | ❑ |
| 1. Did you become emotional more readily? | ❑ | ❑ | ❑ | ❑ | ❑ | ❑ |
| 1. Did it bother you that you become emotional more quickly? | ❑ | ❑ | ❑ | ❑ | ❑ | ❑ |
| 1. Were you depressed or in low spirits? | ❑ | ❑ | ❑ | ❑ | ❑ | ❑ |
| 1. Did you feel that you were a burden to your family and friends | ❑ | ❑ | ❑ | ❑ | ❑ | ❑ |
| 1. Were you afraid to exert yourself? | ❑ | ❑ | ❑ | ❑ | ❑ | ❑ |
| 1. Did you feel limited in takning a trip? | ❑ | ❑ | ❑ | ❑ | ❑ | ❑ |
| 1. Were you afraid of being alone? | ❑ | ❑ | ❑ | ❑ | ❑ | ❑ |

|  | To a great extent | To a high extent | To some extent | To a lesser extent | Not at all | Dont know |
| --- | --- | --- | --- | --- | --- | --- |
| 21) Have you experienced that your expectations, hopes, values ​​or perspectives on life have changed since your blood clot in the lung  ? | ❑ | ❑ | ❑ | ❑ | ❑ | ❑ |
| 22) Are you extra aware of symptoms (heart palpitations, shortness of breath, pain/tenderness in the legs) of a new blood clot in the lung? | ❑ | ❑ | ❑ | ❑ | ❑ | ❑ |

## General health, worries and difficult feelings

*The next question is about your* general health

|  | excellent | Mighty well | well | Less well | Bad |
| --- | --- | --- | --- | --- | --- |
| 23) How do you think your health is overall | ❑ | ❑ | ❑ | ❑ | ❑ |

*In the following comes a series of statements about how you have felt in the past 2 weeks. Tick ​​the box that best describes how often you have experienced or felt the following:*

|  | All the  time | Most of the time | Little over  half of the time | Littke less  than half of the time | Little of the time | At no time |
| --- | --- | --- | --- | --- | --- | --- |
| 24) During the past two weeks**:** |  |  |  |  |  |  |
| 1. Have I been happy and in a good mood | ❑ | ❑ | ❑ | ❑ | ❑ | ❑ |
| 1. Have I felt calm and relaxed | ❑ | ❑ | ❑ | ❑ | ❑ | ❑ |
| 1. Have I felt active and energetic | ❑ | ❑ | ❑ | ❑ | ❑ | ❑ |
| 1. Have I woken up refreshed and rested | ❑ | ❑ | ❑ | ❑ | ❑ | ❑ |
| 1. Has my daily life been filled with things that interest me | ❑ | ❑ | ❑ | ❑ | ❑ | ❑ |

|  | All the  time | Most of the time | Little over  half of the time | Littke less  than half of the time | Little of the time | At no time |
| --- | --- | --- | --- | --- | --- | --- |
| 25) How much of the time during the **last 2 weeks**: |  |  |  |  |  |  |
| a) Have you felt sad or down? | ❑ | ❑ | ❑ | ❑ | ❑ | ❑ |
| b) Have you lacked interest in your daily activity? | ❑ | ❑ | ❑ | ❑ | ❑ | ❑ |
| c) Have you been nervous, tense or felt inner turmoil? | ❑ | ❑ | ❑ | ❑ | ❑ | ❑ |
| d) Been very worried about even the smallest things in your daily life?  ? | ❑ | ❑ | ❑ | ❑ | ❑ | ❑ |

**Blood thinners**

*The next questions concern blood-thinning medication, it can be both as tablets or injections.*

*(If you do not take blood-thinning medication, you must answer not relevant)*

|  | To a great extent | To a high extent | To some extent | To a lesser extent | Not at all | Not relevant |
| --- | --- | --- | --- | --- | --- | --- |
| 26) Do you generally feel satisfied with your blood thinning treatment of your blood clot in the lung? | ❑ | ❑ | ❑ | ❑ | ❑ | ❑ |
| 27) Does it bother you that you may get small bleedings (bruises, bleeding when brushing your teeth, nosebleeds and the like) from your blood thinning treatment? | ❑ | ❑ | ❑ | ❑ | ❑ | ❑ |
| 28) Is it a burden for you taking blood thinning treatment? | ❑ | ❑ | ❑ | ❑ | ❑ | ❑ |
| 29) Do you feel that your blood thinning treatment limits you in your everyday life? | ❑ | ❑ | ❑ | ❑ | ❑ | ❑ |

*(If you are still being treated with blood-thinning medication, you must answer not relevant)*

|  | To a great extent | To a high extent | To some extent | To a lesser extent | Not at all | Ved ikke/ ikke relevant |
| --- | --- | --- | --- | --- | --- | --- |
| 30) Do you feel that you have received the necessary information about why you should stop taking the blood-thinning medication? | ❑ | ❑ | ❑ | ❑ | ❑ | ❑ |
| 31) Are you worried about no longer having to take blood thinners? | ❑ | ❑ | ❑ | ❑ | ❑ | ❑ |

**Thank you for your cooporation!**

**Table S3: Test-retest agreement**

| **Background information** | Kappa, weighted kappa (*) | Lower confidence interval | Upper confidence interval |  |
| --- | --- | --- | --- | --- |
| 02)Number of times diagnosed | 1,00 | - | - |  |
|  |  |  |  |  |
| *03) In the period 6 weeks before the diagnosis with your blood clot in the lung, did you experience any of the following?* |  |  |  |  |
| a) Having broken a hip or lower leg | 1,00 | - | - |  |
| b) Having surgery | 0.83 | 0.34 | 1.00 |  |
| c) Being admitted due to e.g. pneumonia, | 0.46 | 0.00 | 1.00 |  |
| d) Having Covid-19 | 0.00 | - | - |  |
| e) Being admitted for a longer period (more than 3 days) | 0.44 | -0.09 | 1.00 |  |
| f) Been on a flight or long-term seated transport | 1.00 | - | - |  |
| g) Being recently pregnant or having given birth | 1.00 | - | - |  |
| h) Having been in fertility treatment, hormone treatment or taken birth control pills | 1.00 | - | - |  |
| i) Having cancer | 0.77 | 0.00 | 1.00 |  |
| j) Having received chemotherapy | 1.00 |  |  |  |
| k) Having had a blood clot in the leg | 0.44 | -0.12 | 1.00 |  |
| l) None of the above | 0.68 | 0.28 | 1.00 |  |
| m) Do not know | 0,00 |  |  |  |
|  |  |  |  |  |
| *04) In the period 6 weeks before your diagnosis with your blood clot in the lung, did you experience one or more of the following symptoms?* |  |  |  |  |
| a) Shortness of breath | 0.50 | 0.19 | 0.88 |  |
| b) Pain in the chest | 0.64 | 0.00 | 1.00 |  |
| c) tiredness | 0.60 | 0.27 | 0.89 |  |
| d) Dizziness | 0.16 | -0.19 | 0.61 |  |
| e) Heart palpitations | 0.00 |  |  |  |
| f) Fainting | 0.00 |  |  |  |
| g) Pain, swelling, tenderness in the elg | 0.73 | 0.34 | 1.00 |  |
| h) none of the above | 0.48 | -0.09 | 1.00 |  |
| i) Do not know | 0.00 |  |  |  |
|  |  |  |  |  |
| **Information and support** |  |  |  |  |
| *05) Were you comfortable coming home from the hospital after your first  hospitalization with a blood clot in the lung?* | 0.37 | -0.10 | 0.73 |  |
| *06) Did you know what was going to happen after you were discharged from the  hospital? Eg. whether you should contact your own doctor, or whether you would receive a letter from the hospital for a check-up in the ambulatory.* | 0.33 | -0.15 | 0.75 |  |
| *07) Did the healthcare staff use a language that was understandable (not professional language, but comprehensible Danish)?* | 0.27 | -0.03 | 0.64 |  |
| *08) Overall, did you get the oral and written information from the hospital staff that  you needed?* | 0.45 | -0.01 | 0.77 |  |
|  |  |  |  |  |
| *09) Do you feel informed about the following without having to seek out/find the  information yourself:* |  |  |  |  |
| a ) What examinations and treatment courses did you have to go through? | 0.34* | -0.04 | 0.65 |  |
| b) What the disease can mean for your future? | 0.69* | 0.34 | 0.89 |  |
| c) you can do yourself to feel better, e.g. by physical training? | 0.49* | 0.15 | 0.74 |  |
|  |  |  |  |  |
| **Advice, support and offers** |  |  |  |  |
| *10) In connection with your blood clot in the lung, have you been offered the following?:* |  |  |  |  |
| a) How you can best return to the labor market | 0.57 | 0.25 | 0.91 |  |
| b) Physical exercise | 0.32 | 0.00 | 0.65 |  |
| c) Psychological support (therapist course, psychological help) | 0.05 | -0.12 | 0.34 |  |
| d) education about your illness and the blood-thinning medication | 0.31 | -0.02 | 0.63 |  |
| e) Individual education about blood thinners | 0.03 | -0.29 | 0.40 |  |
| f) Education in psychological reactions | 0.27 | -0.16 | 0.79 |  |
| g) Offer of written material describing the disease and the blood-thinning medication | 0.02 | -0.27 | 0.40 |  |
| h) Offer about control scan (CT scan) to see if the blood clot is gone | 0.68 | 0.22 | 1.00 |  |
|  |  |  |  |  |
| *11) Would you have liked the following to have been offered?:* |  |  |  |  |
| a) How you can best return to the labor market | 0.07 | -0.36 | 0.54 |  |
| b) Physical exercise | 0.23 | -0.17 | 0.62 |  |
| c) Psychological support (therapist course, psychological help) | 0.35 | -0.07 | 0.71 |  |
| d) education about your illness and the blood-thinning medication | 0.18 | -0.15 | 0.51 |  |
| e) Individual education about blood thinners | 0.15 | -0.10 | 0.46 |  |
| f) Education in psychological reactions | 0.13 | -0.20 | 0.54 |  |
| g) Offer of written material describing the disease and the blood-thinning medication | 0.05 | -0.28 | 0.37 |  |
| h) Offer about control scan (CT scan) to see if the blood clot is gone | 0.34 | 0.00 | 0.78 |  |
|  |  |  |  |  |
| **PEmb-QOL** |  |  |  |  |
| *12) During the past 4 weeks, how often have you had any of the following symptoms from your lungs? (Circle 1 answer on each line)* |  |  |  |  |
| a) Pain behind or between the shoulder blades? | 0.55 | 0.00 | 1.00 |  |
| b) Pain on or in the chest? | 0.74 | 0.00 | 1.00 |  |
| c) Pain in the back? | 0.49 | -0.04 | 0.85 |  |
| d) Sensation of pressure? | 0.22 | 0.00 | 0.63 |  |
| e) Feeling that there is still “something” there? | 0.43 | 0.00 | 0.81 |  |
| f) “Burning sensation” in the lungs? | 0.18 | 0.00 | 1.00 |  |
| g) “Nagging feeling2 in the lungs? | 0.18 | 0.00 | 0.77 |  |
| h) Difficulty in breathing or breathlessness? | 0.63 | 0.27 | 0.89 |  |
|  |  |  |  |  |
| *13) At what time of day are your lung symptoms most intense? (circle one answer)* | 0.64 | 0.24 | 0.89 |  |
| *14) Compared to 1 year ago, how would you rate the condition of your lungs in general now? (circle one answer)* | 0.58 | 0.26 | 0.87 |  |
|  |  |  |  |  |
| *15) The following items are about activities that you might do in a typical day.  Do your lung symptoms now limit you in these activities? If so, how much?* |  |  |  |  |
| a) **Daily activities at work** | 0.79 | 0.49 | 1.00 |  |
| b) **Daily activities at home** (e.g. housework, ironing,doing odd jobs/repairs around the house, gardening etc…) | 0.70 | 0.41 | 1.00 |  |
| c) **Social activities** (Such as traveling, going to the cinema, parties, shopping) | 0.89 | 0.66 | 1.00 |  |
| d)**Vigorous activities,** such as running, lifting heavy objects, participating in strenuous sports | 0.48 | 0.16 | 0.75 |  |
| e) **Moderate activities,** such as moving a table, hoovering, swimming or cycling | 0.82 | 0.49 | 1.00 |  |
| f) Lifting or carrying groceries | 0.89 | 0.46 | 1.00 |  |
| g) Climbing **several** flights of stairs | 0.68 | 0.41 | 1.00 |  |
| h) Climbing **one** flight of stairs | 0.51 | 0.00 | 1.00 |  |
| i) Bending, kneeling squatting | 0.03 | -0.27 | 0.34 |  |
| j) Walking **more than half a mile** | 0.44 | 0.16 | 0.73 |  |
| k) Walking **a couple of hundred years** | 0.68 | 0.00 | 1.00 |  |
| l) Walking **about one hundred yeards** | 0.60 | -0.08 | 1.00 |  |
| m) Washing or dressing yourself | 0.00 |  |  |  |
|  |  |  |  |  |
| *16) During the past 4 weeks, have you had any of the following problems with your  work or other regular daily activities as a result of your lung symptoms?* |  |  |  |  |
| a) Cut down the **amount of time** you spent on work or other activities | 0.51 | 0.00 | 0.83 |  |
| b) **Accomplished** less than you would like | 0.66 | 0.32 | 1.00 |  |
| c) Were limited in the **kind** of work or other activities | 0.38 | -0.14 | 0.83 |  |
| d) Had **difficulty** performing the work or other activities (e.g. it took extra effort) | 0.56 | 0.21 | 0.88 |  |
|  |  |  |  |  |
| *17) During the past 4 weeks, to what extent have your lung symptoms interfered with your normal social activities with family, friends, neighbours or groups?* | 0.46 | 0.12 | 0.75 |  |
| *18) How much pain around your shoulder blades/pain in your chest have you experienced during the past 4 weeks?* | 0.77 | 0.51 | 1.00 |  |
| *19) How much breathlessness have you experienced in the past 4 weeks?* | 0.28 | -0.01 | 0.56 |  |
|  |  |  |  |  |
| 20) *How much of the time during the past 4 weeks?* |  |  |  |  |
| a) Were you worried about having another pulmonary embolism? | 0.40 | 0.00 | 0.73 |  |
| b) Did you feel irritable? | 0.18 | -0.14 | 0.53 |  |
| c) Would you have been worried if you had to stop taking anticoagulant medication? | 0.39 | 0.09 | 0.72 |  |
| d) Did you become emotional more readily? | 0.43 | 0.01 | 0.62 |  |
| e) Did it bother you that you become emotional more quickly? | 0.41 | -0.10 | 0.57 |  |
| f) Were you depressed or in low spirits? | 0.19 | 0.00 | 0.47 |  |
| g) Did you feel that you were a burden to your family and friends | 0.43 | -0.02 | 0.62 |  |
| h) Were you afraid to exert yourself? | 0.51 | 0.14 | 0.92 |  |
| i) Did you feel limited in takning a trip? | 0.67 | 0.25 | 0.85 |  |
| j) Were you afraid of being alone? | 0.44 | -0.12 | 1.00 |  |
|  |  |  |  |  |
| *21) Have you experienced that your expectations, hopes, values ​​or perspectives on life  have changed since your blood clot in the lungs* | 0.33 | 0.06 | 0.70 |  |
| *22) Are you extra aware of symptoms (heart palpitations, shortness of breath, pain/tenderness in the legs) of a new blood clot in the lung?* | 0.38 | 0.00 | 0.66 |  |
|  |  |  |  |  |
| **General health: WHO5, ASS-2, MDI-2** |  |  |  |  |
| *23) How do you think your health is overall* | 0.77 | 0.58 | 0.93 |  |
| *24) During the past two weeks****:*** |  |  |  |  |
| a) Have I been happy and in a good mood | 0.45 | 0.02 | 0.63 |  |
| b) Have I felt calm and relaxed | 0.23 | -0.05 | 0.53 |  |
| c) Have I felt active and energetic | 0.35 | 0.03 | 0.58 |  |
| d) Have I woken up refreshed and rested | 0.54 | 0.14 | 0.69 |  |
| e) Has my daily life been filled with things that interest me | 0.60 | -0.06 | 0.80 |  |
|  |  |  |  |  |
| *25) How much of the time during the* ***last 2 weeks****:* |  |  |  |  |
| a) Have you felt sad or down? | 0.43 | 0.17 | 0.72 |  |
| b) Have you lacked interest in your daily activity? | 0.58 | 0.30 | 0.91 |  |
| c) Have you been nervous, tense or felt inner turmoil? | 0.36 | 0.10 | 0.60 |  |
| d) Been very worried about even the smallest things in your daily life? | 0.63 | 0.19 | 0.90 |  |
|  |  |  |  |  |
| **Anticoagulation therapy** |  |  |  |  |
| *Treated* |  |  |  |  |
| *26) Do you generally feel satisfied with your blood thinning treatment of your blood clot in the lung?* | 0.63 | 0.40 | 0.79 |  |
| *27) Does it bother you that you may get small bleedings (bruises, bleeding when brushing your teeth, nosebleeds and the like) from your blood thinning treatment?* | 0.64 | 0.41 | 0.83 |  |
| *28) Is it a burden for you taking blood thinning treatment?* | 0.69 | 0.45 | 0.89 |  |
| *29) Do you feel that your blood thinning treatment limits you in your everyday life?* | 0.73 | 0.49 | 1.00 |  |
| *Not treated* |  |  |  |  |
| 30) Do you feel that you have received the necessary information about why you should stop taking the blood-thinning medication? | 0.83 | 0.59 | 1.00 |  |
| 31) Are you worried about no longer having to take blood thinners? | 0.74 | 0.50 | 1.00 |  |

Commonly accepted interpretations of kappa statistics state that values 0.81-1 represent almost perfect agreement; 0.61-0.80 substantial agreement; 0.41-0.60 moderate agreement; 0.21-0.40 fair agreement; 0.01-0.20 slight agreement; and <0 less than chance agreement. For intraclass correlation coefficients, large values (close to 1) indicate low random error variability and thus high agreement. Slight agreement (≤ 0.20): 19.8%, Fair agreement (% 0.21-0.40): 18.9%, Moderate agreement (% 0.41-0.60): 28.3%, Substantial agreement (% 0.61-0.80): 21.7%, Perfect agreement (%> 0.80): 11.3%
